# Dynamics of an Infectious Keratoconjunctivitis Outbreak by *Mycoplasma conjunctivae* on Pyrenean Chamois *Rupicapra p. pyrenaica*

**DOI:** 10.1371/journal.pone.0061887

**Published:** 2013-04-24

**Authors:** MaríaCruz Arnal, Juan Herrero, Christian de la Fe, Miguel Revilla, Carlos Prada, David Martínez-Durán, Ángel Gómez-Martín, Olatz Fernández-Arberas, Joaquín Amores, Antonio Contreras, Alicia García-Serrano, Daniel Fernández de Luco

**Affiliations:** 1 Departamento de Patología Animal, Facultad de Veterinaria, Universidad de Zaragoza, Zaragoza, Spain; 2 Área de Ecología, Departamento de Ciencias Agrarias y Medio Natural, Escuela Politécnica Superior de Huesca, Universidad de Zaragoza, Huesca, Spain; 3 Departamento de Sanidad Animal, Facultad de Veterinaria, Universidad de Murcia, Murcia, Spain; 4 Ega Wildlife Consultants, Zaragoza, Spain; Université de Sherbrooke, Canada

## Abstract

Between 2006 and 2008, an outbreak of Infectious Keratoconjunctivitis (IKC) affected Pyrenean chamois *Rupicapra p. pyrenaica*, an endemic subspecies of mountain ungulate that lives in the Pyrenees. The study focused on 14 mountain massifs (180,000 ha) where the species’ population is stable. Cases of IKC were detected in ten of the massifs and, in five of them, mortality was substantial. The outbreak spread quickly from the first location detected, with two peaks in mortality that affected one (2007) and three (2008) massifs. In the latter, the peak was seasonal (spring to autumn) and, in the former, the outbreak persisted through winter. To identify the outbreak’s aetiology, we examined 105 Pyrenean chamois clinically affected with IKC. TaqMan rt-PCR identified *Mycoplasma conjunctivae* in 93 (88.5%) of the chamois. Another rt-PCR detected *Chlamydophila spp*. in 14 of chamois, and 12 of those had mixed infections with mycoplasmas. In the period 2000–2007, the chamois population increased slightly (λ 1.026) but decreased significantly during the IKC outbreak (λ 0.8, 2007–2008; λ 0.85, 2008–2009) before increasing significantly after the outbreak (λ 1.1, 2009–2010). Sex-biased mortality shifted the adult sex ratio toward males (from 0.6 to 0.7 males per female) and reduced productivity slightly. Hunting was practically banned in the massifs where chamois experienced significant mortality and allowed again after the outbreak ended. Long-term monitoring of wild populations provides a basis for understanding the impacts of disease outbreaks and improves management decisions, particularly when species are subject to extractive exploitation.

## Introduction

Infectious keratoconjunctivitis (IKC) is a contagious disease that is common in domestic ruminants and wild *Caprinae*. Domestic sheep can act as reservoir hosts and are the probable source of infections in wildlife, for which the impact of IKC is comparatively higher [1], [2].

Several microorganisms including *Chlamydophila* and *Mycoplasma* species are suspected IKC aetiological agents [3], [1]. *Mycoplasma conjunctivae* occurs in wild ruminants, and is known to affect Alpine ibex *Capra ibex*, Alpine chamois *Rupicapra r. rupicapra* [4], [5], [6], Pyrenean chamois *Rupicapra p. pyrenaica* [7], European mouflon *Ovis aries* [8], and Himalayan thar *Hemitragus jemlaicus* [9]. *Chlamydophila spp.* was the causative organism in outbreaks of IKC in bighorn sheep *Ovis canadensis* [10], and was isolated in IKC-affected mule deer *Odocoileus hemionus* [11]. Other bacteria such as *Moraxella ovis*, *Corynebacterium pyogenes*, *Rickettsia conjunctivae,* and *Staphyloccocus aureus* have been isolated from animals that were affected with IKC [12], [5]. However, *Mycoplasma conjunctivae* is considered the primary cause of IKC in wild *Caprinae* [5].

Multiple outbreaks of IKC in Alpine ibex and Alpine chamois populations have been described in the literature [4], [5], [6], [13]. In Spain, the first reported cases of IKC in wild *Caprinae* occurred in 1952 in Pyrenean chamois and in 1979 in Cantabrian chamois *Rupicapra pyrenaica parva*. Other outbreaks of IKC occurred in the Pyrenees in 1982 and 1992 [14], [15]. In the 1982 outbreak, the pathogen *Chlamydia psittaci* was isolated from a single affected animal [16]; however, the principal etiological agent was never identified. In that outbreak, most of the subpopulations of Pyrenean chamois were affected. Considering that there was no population monitoring, approximate estimations considered a decrease in 50% of total animals. Almost 30 yr later, a new outbreak of IKC affected populations of Pyrenean chamois, which have been subjected to long-term monitoring [17], [18], [19], and has provided an opportunity to quantify the effects of the outbreak on Pyrenean chamois.

The aims of the study were (i) to describe the spread of IKC in the 2006–2008 outbreak and its effects on the population of Pyrenean chamois, (ii) to determine whether the aetiology of the IKC outbreak was associated with the presence of mycoplasmas or *Chlamydophila spp.*, (iii) to assess the occurrence of *M. conjunctivae* in domestic and other wild ruminants in the area, and (iv) to describe the lesions associated with the infection.

## Materials and Methods

### Study Area

**Figure 1:**
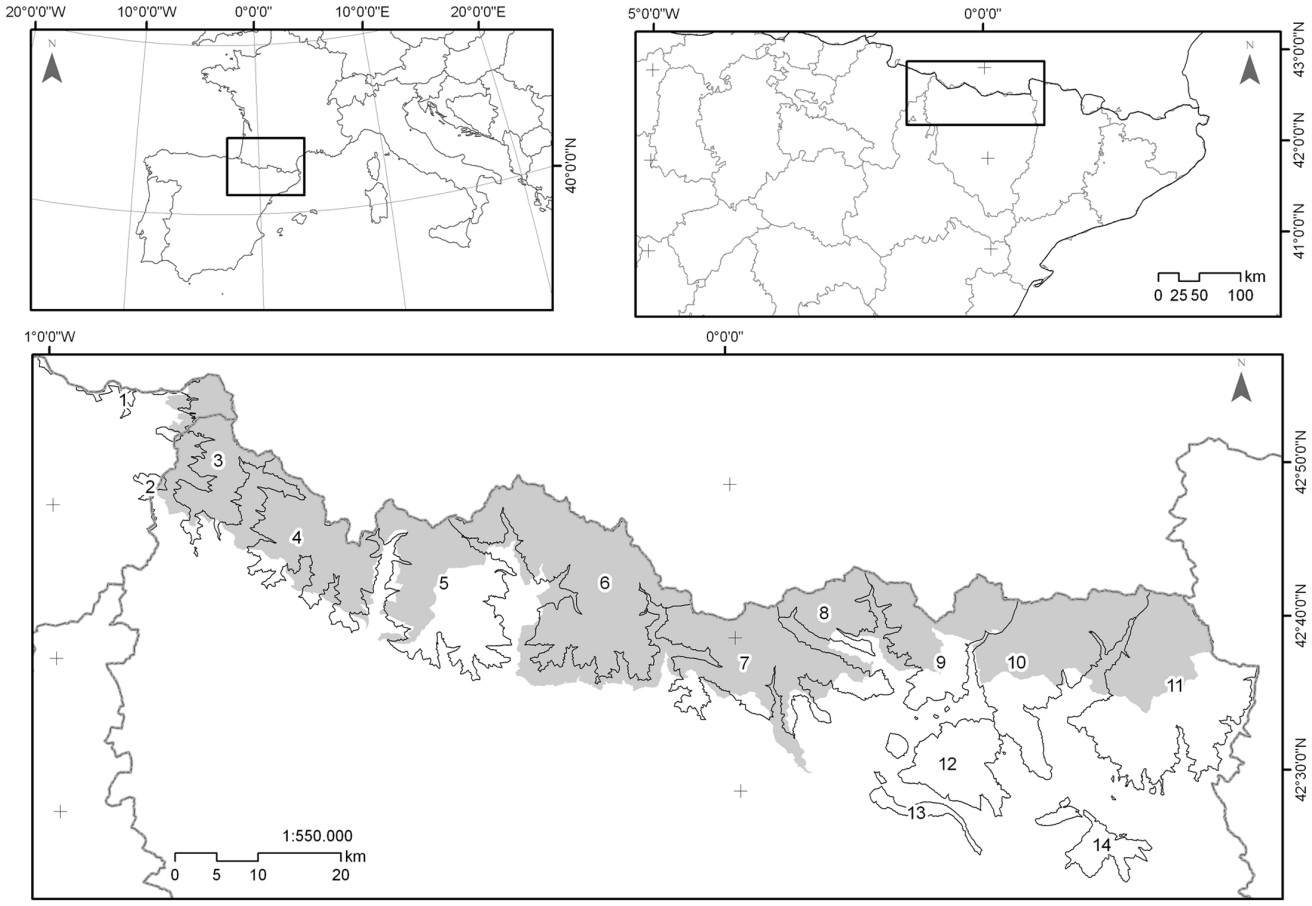
Study area. Mountain massifs in the study area within the Spanish Pyrenees. 1. Ori; 2. Ezkaurre; 3. Larra – Peña Forca; 4. Bixaurín; 5. Anayet; 6. Biñamala; 7. Monte Perdido; 8. Liena; 9. Punta Suelsa; 10. Posets; 11. Maladeta; 12. Cotiella; 13. Sierra Ferrera; 14. Turbón. Grey indicates the area directly managed by the regional administration in Aragon.

The study area comprised the entire distribution area of the subspecies in Aragon and Navarre (Spanish Pyrenees), which is divided into 14 natural management units (massifs); 10 in the Pyrenees (North) and 4 in the Pre-Pyrenees (South). Among the massifs (180,000 ha) there are 50 hunting grounds (which are managed by local hunters), a National Park and a Nature Reserve (where hunting is forbidden) and five Game Reserves (GR) that are managed by the Government of Aragon (Figure 1). Massifs are the planimetrical surfaces above 1,600 m in mountains that are >2,000 m high, which is the area of biological significance for the species that has seasonal vertical migrations in the Pyrenees [20], [21]. The highest elevation is 3,404 m. The climate is influenced by the relief. Mean annual T is <12°C, average T is <0°C at 3,000 m and 11°C at 600 m. In the high western valleys, where there is an Atlantic influence, annual precipitation is >2,000 mm; in the east, precipitation is <1,000 mm, and a significant proportion falls as snow. Biogeographically, the area is within the Eurosiberian region, with some areas that are transitional to the Mediterranean. Subalpine pastures occur between 1,600–2,000 m, in areas that are dominated by forests of Scots pine *Pinus sylvatica* and mountain pine *Pinus uncinata*. In the montane habitats below 1,600 m, European beech *Fagus sylvatica* and silver fir *Abies alba* predominate. In the lowest forests, holm oak *Quercus ilex* and white oak *Quercus humilis* predominate, along with pastures.

Other important wild ungulates in the area include wild boar *Sus scrofa*, roe deer *Capreolus capreolus*, and red deer *Cervus elaphus* (in order of density and distribution) [22], [23]. Golden eagle *Aquila chrysaetos* and red fox *Vulpes vulpes* are the most important predators. Hunting quotas are based on population estimates [18], and about 500 adult chamois (1∶1 sex ratio, SR) are hunter-harvested each year. In the area, the human density is about 4 inhabitants km^−2^. The main economic activities are tourism and livestock farming.

### Ethics Statement

This study on free-ranging chamois was performed with the permission and support of local authorities (Aragon Government) to update information on the demographic and health status of current populations of game species in the Aragonese jurisdiction, the Hunting Law 5/2002. All samples were collected from legally hunted Pyrenean chamois during the hunting season (hunting quotas are annually established by the Department of Agriculture, Livestock and Environment of the Aragon Government) and from animals that died or were ill and euthanized by intravenous injection of sodium pentobarbital. The collection of this material did not require the approval of the Ethics Committee for Animal Experimentation because they are considered routine veterinary practice without experimentation.

In Navarre, Pyrenean chamois are categorized as Vulnerable, and local authorities allow demographic and sanitary monitoring.

In coordination with the Department of Agriculture, Livestock and Environment of the Aragon Government, sheep samples were also collected in chamois areas in collaboration with sheep owners.

### Demographic Procedures

In Aragon and Navarre (Spain), populations of Pyrenean chamois are monitored using total counts above the timberline based on itineraries [24] performed in June or July and November. The first is used to estimate productivity and the latter to estimate the adult SR. In one of the massifs, animals were counted in April because of the low elevations and scarce grasslands in areas where chamois occupy open areas. During surveys, individuals were identified as adult males, adult females, yearlings (1–2 yr of age), kids, or undetermined. Above 1,600 m, the area was divided into natural management units (massifs) (Figure 1). Above the timberline, total counts were performed each year in the small massifs and every five years in the large ones. In the latter, in years without a complete survey, an annual selection of fixed itineraries was made to provide representative estimates of the population’s demographic parameters, structure, and trends, because they included at least 50% of the number of individuals seen in the complete counts. Adult SR was calculated dividing adult males per adult females and productivity dividing kids per female. Since 1995, when the first surveys were performed, the number of large massifs that were surveyed using global total counts, rather than annual selected itineraries, increased gradually and, since 2008, all of the massifs were surveyed fully each year. Professional rangers performed most of the counts, with the assistance of hunters, technicians, local volunteers, and the authors, using binoculars, spotting scopes, and maps of the itineraries, upon which the locations of groups were recorded.

Since 2006, rangers at the GR and Ordesa and Monte Perdido National Park filed bi-monthly reports of the number of chamois found dead, some of which were collected for necropsy.

### Animals and Sample Collection

#### IKC-affected chamois

Between 2006 and 2008, 105 Pyrenean chamois clinically affected with IKC were examined (1 in 2006, 32 in 2007, and 72 in 2008). Systematic necropsies were performed on 24 dead animals and 81 ill animals which were euthanized after inspection by a qualified veterinarian. Samples of the eye, the third eyelid, eyelids, lung, liver, spleen, kidney, and central nervous system were collected and fixed in 10% neutral buffered formalin and embedded in paraffin. Tissue sections (5 µm) were stained with Haematoxilin-Eosine (HE) for histopathological analyses.

#### Healthy wild ruminants

To assess the prevalence of *M. conjunctivae* from apparently (clinically) healthy chamois, 190 eye swabs were collected in the GR during the hunting season (April-May and September-December) over a four-year period between 2006 and 2009. Consequently, we analyzed samples collected during (n = 108) and after (n = 82) the outbreak. In addition, in 2008, eye swabs were collected from 33 roe deer and 14 red deer hunter-harvested in GR, none of which had signs of IKC. Swabs samples from hunter-harvested animals were taken from behind the third eyelid of each eye, and immediately frozen at −20°C.

#### Sheep

In 2008 and 2009, respectively, livestock censuses indicated that there were 206 and 199 sheep owners in the study area. In those years, 67,163 and 70,577 domestic sheep spent the spring, summer, and autumn in Pyrenean meadows, some in mixed flocks (mixing of sheep between flocks). In the meadows, most (64.7%) of those flocks contained 20–850 small ruminants and the others (35.3%) had 1,200–2,700 animals. To verify the presence of *M. conjunctivae* in domestic sheep, in 2008 and 2009, respectively, 17 and 13 flocks from the study area were selected. In autumn, almost 20 apparently (clinically) healthy animals from each flock were sampled. A total of 617 animals were sampled, 349 in 2008 and 268 in 2009. Conjunctival eye swabs were collected from each eye, and immediately frozen at −20°C.

### DNA Extraction

Conjunctival swabs were placed into microcentrifuge tubes that contained 0.5 ml of lysis buffer (100 mM Tris.HCl pH 8.5, 0.05% Tween 20, and 0.24 mg/ml proteinase K) and mixed using the vortex for 1 min at room temperature. The swabs were removed and, to obtain the lysates for the PCR reactions, the buffer was incubated at 60°C for 60 min and at 95°C for 15 min. The DNA from the tissue was extracted from 25 µg of each sample using the High Pure PCR Template Preparation Kit (Roche Diagnostics) in accordance with manufacturer’s instructions.

### PCR and Real-time PCR Techniques

#### Mycoplasma spp

To detect *M. conjunctivae*, an *lppS*-based TaqMan real-time PCR was initially performed [25] by using 2 µl of test sample, including 900 nM of primers LPPS-TM-L, LPPS-TM-R, and 300 nM of probe LPPS-TM-FT and TaqMan Universal PCR Master Mix No AmpErase UNG (Applied Biosystems) in 20 µl volume in the presence of an exogenous internal positive control (Applied Biosystems) that verified amplification. The PCR reactions were run on a 7500 Fast real-time PCR system (Applied Biosystems) using the standardized parameters and the presence/absence endpoint assays. The amplification products were detected using the 7500 Software v2.0.3 Fast Systems (Applied Biosystems).

In addition, a conventional PCR for detecting *Mycoplasma* spp. [26] and specific detection assays for *M. agalactiae* [27] and *Mycoplasma mycoides* cluster members [28] were also performed in a total volume of 25 µl. Each amplification reaction mixture was prepared containing 200 mM of each deoxynucleoside triphosphate, 10 mM Tris-HCl, 2 mM MgCl2, 2 U Taq DNA polymerase (Bio Line, Barcelona, Spain), 0.2 mM of each corresponding primer pair, and 8 µl of template DNA. Amplification was performed in a DNA thermal I-cycler (Bio-Rad, Hercules, CA, USA). The PCR amplification products were run by gel electrophoresis on 1% (wt/vol) agarose gels and visualized using a UV transilluminator (Syngene, Frederick, MD, USA) after ethidium bromide staining.

#### Chlamydophila spp

We developed a *Chlamydophila* genus-specific real-time PCR that amplified a conserved region of 16S rRNA. All of the *Chlamydophila* 16S rRNA regions in the GenBank database were included in the design. We designed the primers Chl16S880F (5′-TATGCCGCCTGAGGAGTACAC; nucleotide (nt) 880–900) and Chl16S959R (5′-ACAAGCAGTGGAGCATGTGG; nt 940–959), and the TaqMan MGB probe Chl16S904P (5′-FAM-CAAGGGTGAAACTC-MGB; nt 904–917) using the Primer Express software 3.0 (Applied Biosystems, Foster City, CA, USA). The primers amplify an 80 bp fragment of the conserved region of 16S rRNA. The specificity of the oligonucleotides was confirmed using BLAST-N software (NCBI; www.ncbi.nlm.nih.gov). The assays used 2 µl of test sample, 900 nM of each primer, and 200 nM of probe in a 20 µl reaction mixture volume that contained Universal PCR Master Mix No AmpErase UNG (Applied Biosystems). An exogenous internal positive control (Applied Biosystems) was introduced into each reaction well. The DNA was amplified using the following cycling parameters: heating at 50°C for 2 min and at 95°C for 10 min, followed by 40 cycles of a two-stage temperature profile of 95°C for 15 s and 60°C for 1 min. The cycle threshold value (Ct) was calculated automatically.

The detection and quantification limits of the PCR assays were identified using genomic DNA isolated from the *Chlamydophila abortus* strain AB7. The standard curve (Ct values vs. log DNA copies) generated by eight serial dilutions of *C. abortus* was linear with an R^2^ value of 0.9983, a slope of −3.5221, and the efficiency was very close to 2 (100% efficiency).

### Statistical Analysis

Changes in the annual number of chamois as a function of time were analyzed using Poisson regression [29], [30], in which the dependent variable is a count that follows the Poisson distribution. We defined a generalized linear model fit with the GLM procedure of R [31] with the response variable given by the annual count of chamois, a Poisson error distribution, and a natural log link function for the exponential growth model for which the parameter b can be interpreted as r, the intrinsic rate of increase, and lambda λ comes from the r neperian antilog.

We applied a logistic regression to data regarding presence or absence of *M. conjunctivae* in the sheep flocks in order to study the relation of this variable with the variables year, massif and flock. We used the Backward Stepwise method considering the likelihood ratio.

Means were compared using T test after the variables were tested for normality. We also used χ^2^ test. These analyses were performed using SPSS (SPSS inc., Chicago, Illinois, USA).

## Results

### Outbreak Description

**Figure 2:**
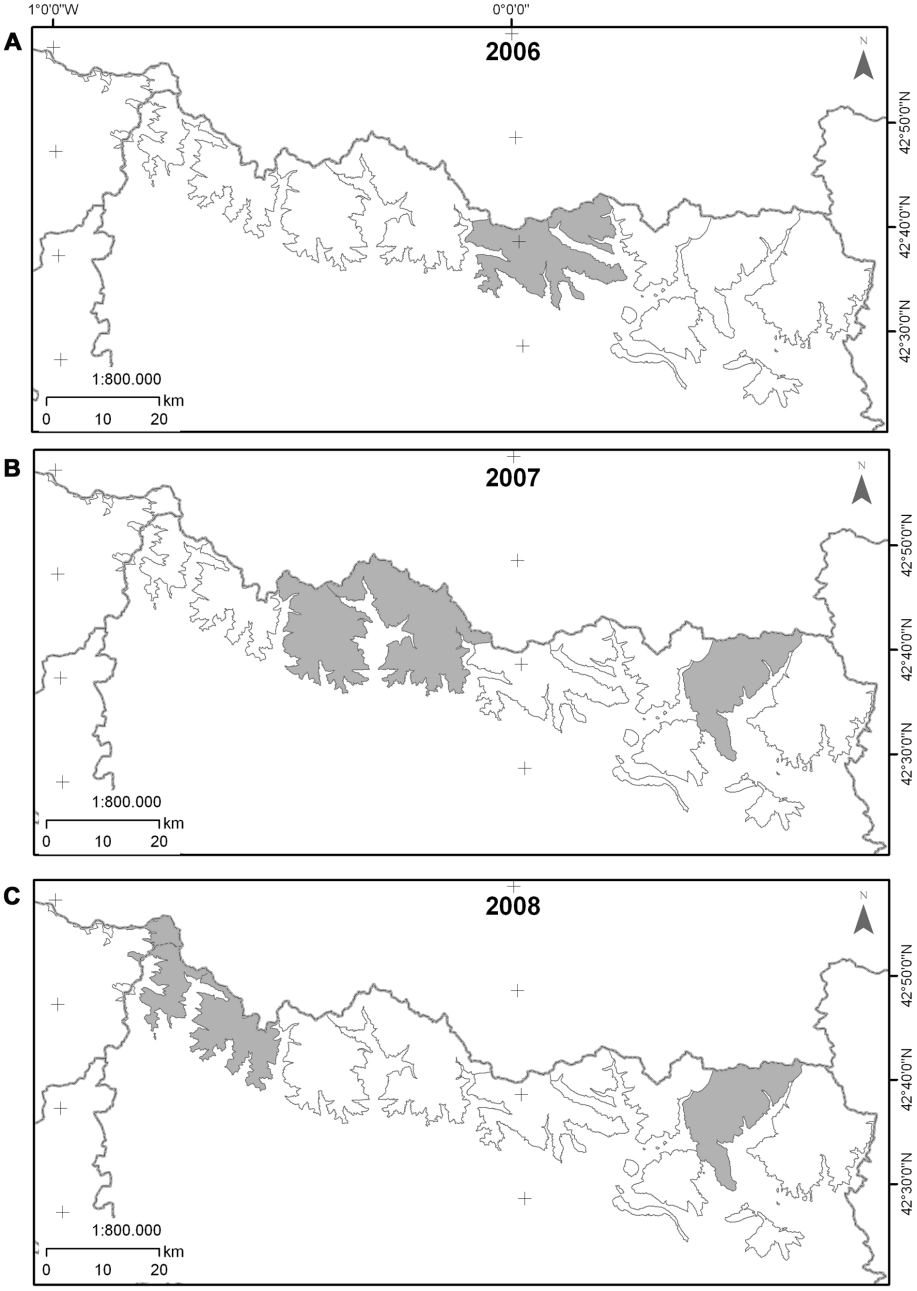
Spatial distribution over Pyrenean massifs of IKC-outbreak locations for years: A) 2006, B) 2007 and C) 2008.

In summer 2006, an outbreak was detected in Ordesa and Monte Perdido National Park within Monte Perdido massif. By autumn 2006, it had spread eastward into Liena and, by summer 2007 reached Posets, where it persisted until autumn 2008. By spring 2007, the outbreak had spread westward to Biñamala. By summer 2007, the disease reached Anayet and, by spring 2008, chamois in Bixaurín and Larra-Peña Forca, the western limit of population, were affected. Stable populations in the Pre-Pyrenean massifs were not affected (Ezcaurre, Cotiella, Sierra Ferrera and Turbón) (Figure 2). Although sporadic cases were observed in the affected massifs, until autumn 2008, the outbreak did not recur in subsequent years and only lasted more than a year in Posets.

### Population Trend

**Figure 3:**
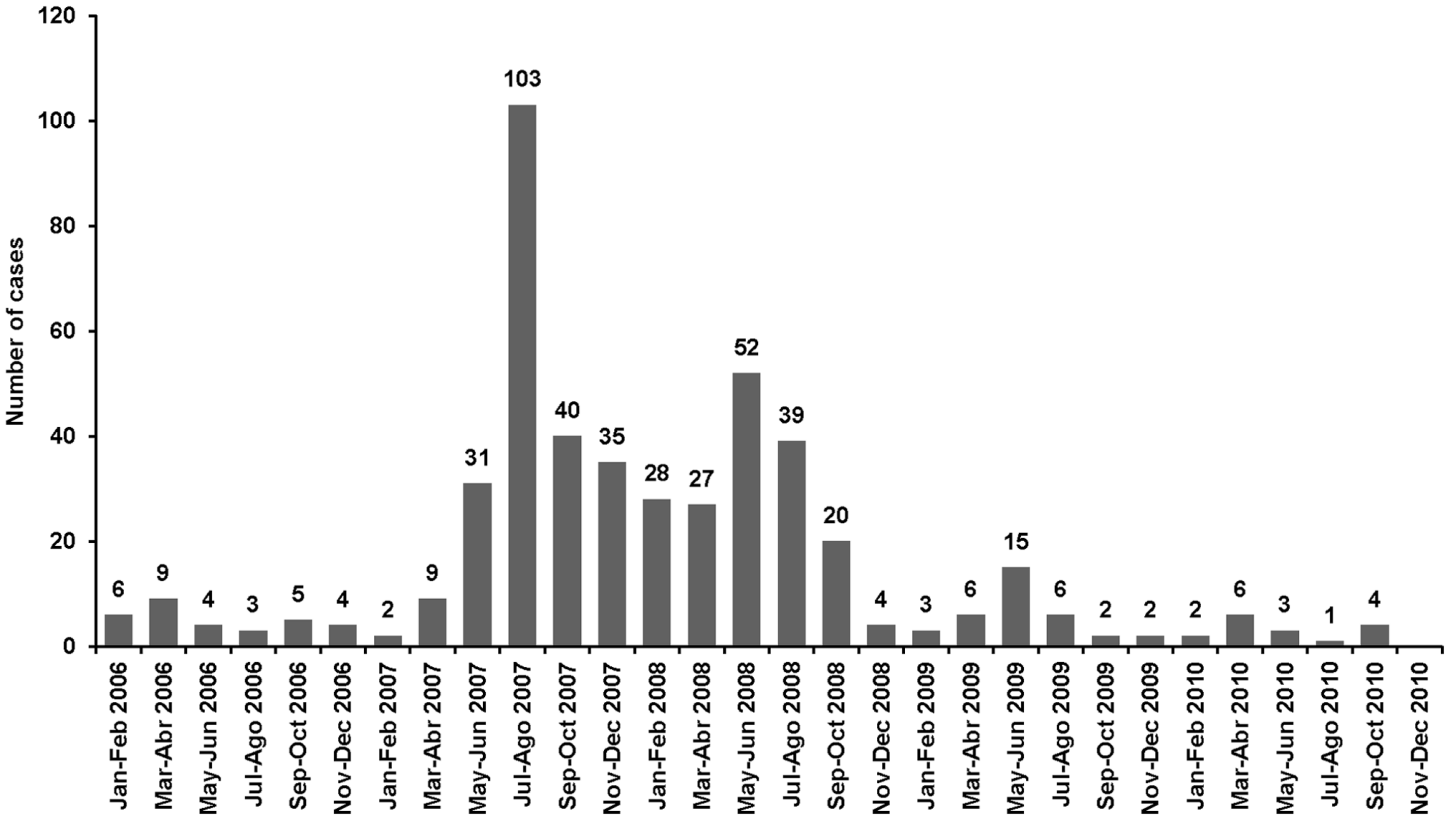
Pyrenean chamois found dead in the Aragonese and Navarrese Pyrenees.

In the period 2000–2007, the chamois population increased slightly (λ 1.0301; 95% CI: 1.0275–1.0328; *p*<0.001) but it decreased significantly during the IKC outbreak (λ 0.8586; 95% CI: 0.8482–0.8691; *p*<0.001, 2007–2009), before increasing significantly after the outbreak (λ 1.1138; 95% CI: 1.0861–1.1422; *p*<0.001, 2009–2010) (Figure 3).

### Changes in SR, Productivity, and Grouping

The average SR in the periods 2000–2007 (0.59; n = 8; SE = 0.00964; 95% CI: 0.5722–0.6178) and 2008–2010 (0.717; n = 3; SE = 0.00882; 95% CI: 0.6787–0.7546) differed significantly (t = −7.2; df = 9; *p*<0.0001) by 0.122 (SE = 0.017). The SR was between 0.08 and 0.16 higher after the outbreak.

Average productivity between 2000 and 2007 (0.708; n = 8; SE = 0.0144) was higher than it was between 2008 and 2010 (0.65; n = 3; SE = 0.03), and the difference was marginally statistically significant (t = 2, df = 9; *p* = 0.082). During the outbreak, individuals were observed as a singleton kid, kids with adult males only, and females with two or three kids.

### Chamois Found Dead

**Figure 4:**
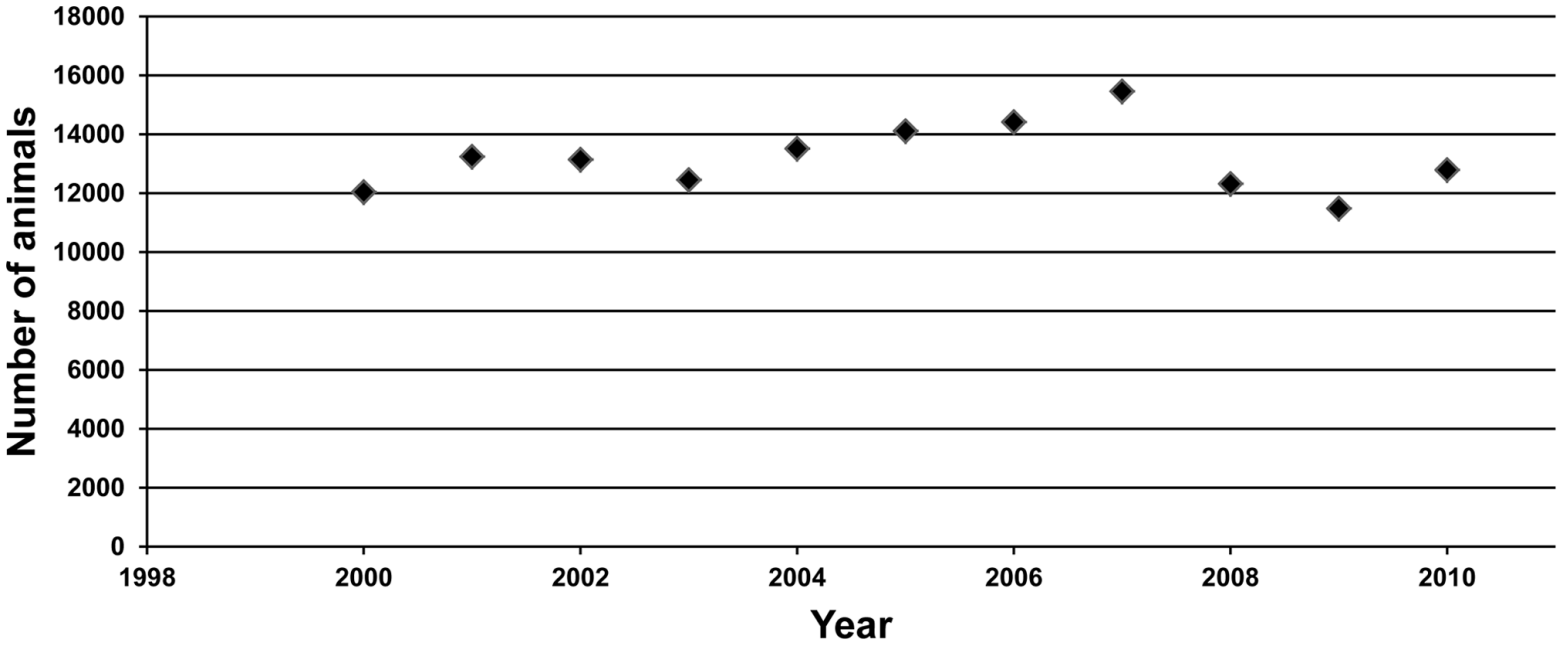
Trend of Pyrenean chamois in the Aragonese and Navarrese Pyrenees.

Between 2006 and 2010, in the bi-monthly reports, 471 chamois were found dead within the study area (Figure 4). In 2010, only 16 chamois were found dead; however, in 2007 and 2008 mortality was substantial. About half of the deaths were recorded in 2007 (n = 220), particularly in summer (n = 103) and primarily in Biñamala (n = 93). In 2008 (n = 170), most of the dead animals were found in Larra-Peña Forca, Bixaurín, Anayet, and Posets. In Posets, the disease persisted between autumn 2007 and autumn 2008. Among the dead animals in these two years (2007–2008, n = 272), 66% were females and 34% were males, age was described by rangers in 316 animals, 70% were adults, 20% were yearlings, and 10% were kids (Table 1).

**Table 1:** Age and sex of chamois found dead in the Aragonese Pyrenees (N = 471, 2006–2010).

			Age			
Year	Sex	0	1	< = 2	Unknown	Total
2006	Female		1	3	3	7
	Male	2		7		9
	Unknown	4	4	1	6	15
		**6**	**5**	**11**	**9**	**31**
2007	Female		7	95	12	114
	Male	1	2	48	9	60
	Unknown	18	24		4	46
		**19**	**33**	**143**	**25**	**220**
2008	Female	1	11	45	9	66
	Male	1	4	21	6	32
	Unknown	12	14	12	34	72
		**14**	**29**	**78**	**49**	**170**
2009	Female		2	9	2	13
	Male		2	7	1	10
	Unknown	2	2	2	5	11
		**2**	**6**	**18**	**8**	**34**
2010	Female	2		3	1	6
	Male			5		5
	Unknown	1	1	1	2	5
		**3**	**1**	**9**	**3**	**16**
	**Total**	**44**	**74**	**259**	**94**	**471**

### IKC-affected Chamois

#### Macroscopic and microscopic lesions

**Figure 5:**
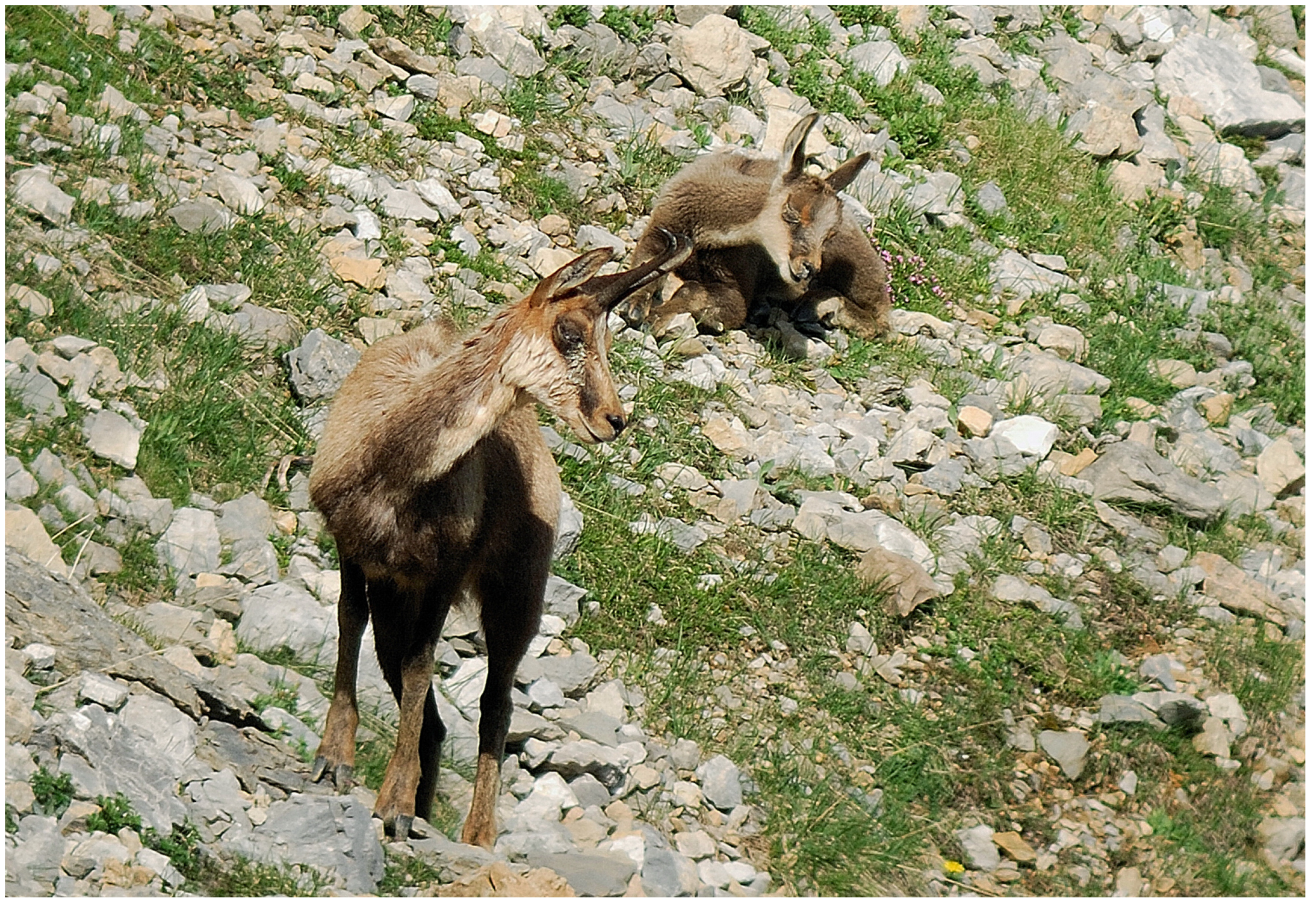
Adult female and kid affected with IKC.

**Figure 6:**
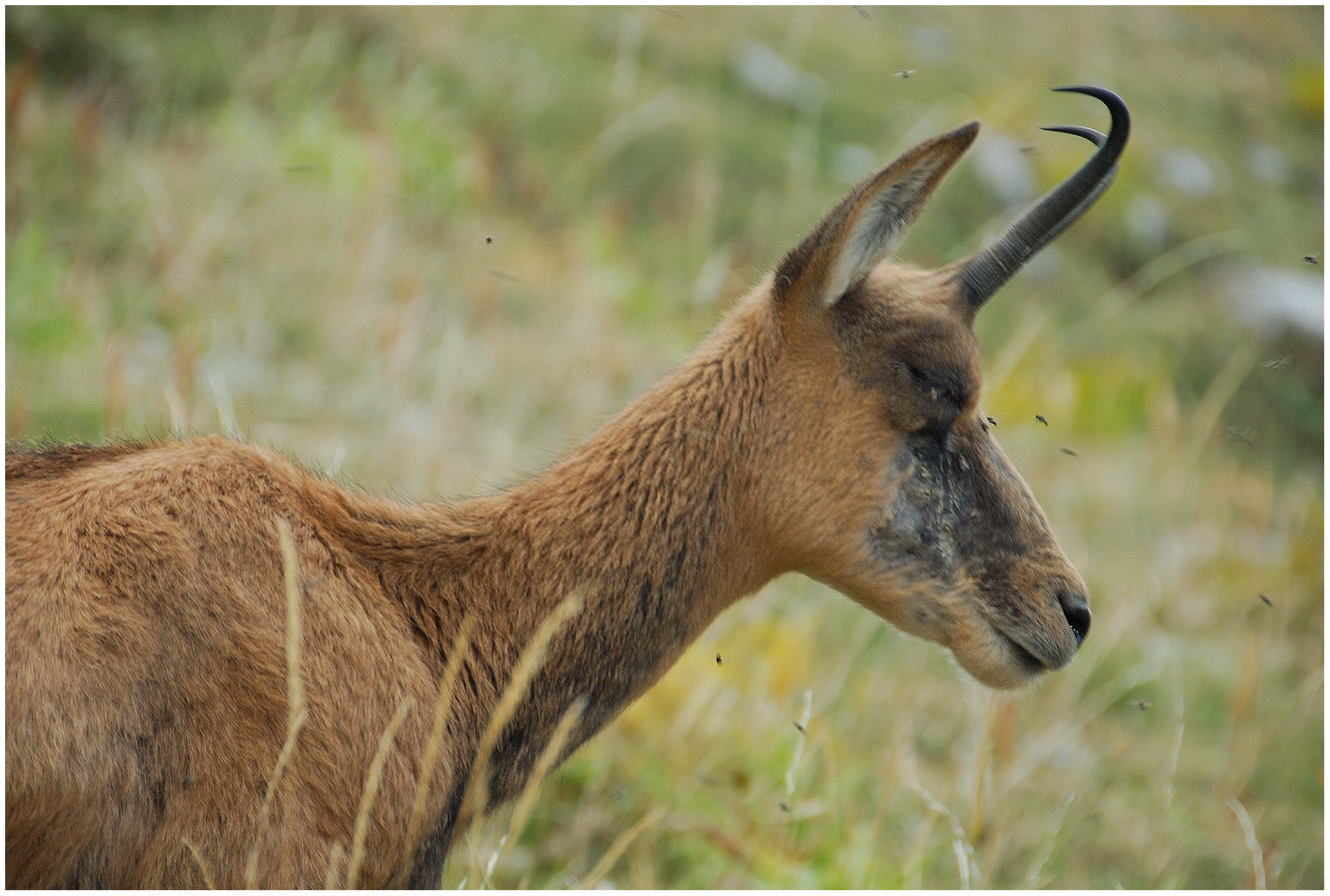
IKC-affected female whit many flies around the head.

In this study, 105 Pyrenean chamois were analysed (Table 2). Eighty-one (77.1%) of them were euthanized after inspection and exhibited behavioural changes such as circling, disorientation, stumbling, and signs of blindness (Figure 5–6). Another 24 chamois (22.8%) were found dead with signs of IKC. Among the dead animals, four were found drowned in mountain lakes, four had wounds consistent with dog bites, four had polytrauma, one had enterotoxaemia and three had been partially eaten after death. Eight of the 24 had no other injuries. The SR among the analysed animals (24.8% males, 75.2% females) was significantly more female-biased than the population before the outbreak (0.59, 2000–2007; χ^2^ 6.9; df = 1; *p* = 0.009). No IKC-affected animals were found in 2009 and 2010.

**Table 2:** Age and sex of chamois analyzed in the Aragonese and Navarrese Pyrenees with infectious keratoconjunctivitis during the outbreak (N = 105, 2006–2008).

	Found dead		Euthanized		Total
Age	Females	Males	Females	Males	
Adults	16	8	54	13	91
Yearlings	0	0	5	2	7
Kids	0	0	4	3	7
**Total**	**16**	**8**	**63**	**18**	**105**
	**24**		**81**		

**Figure 7:**
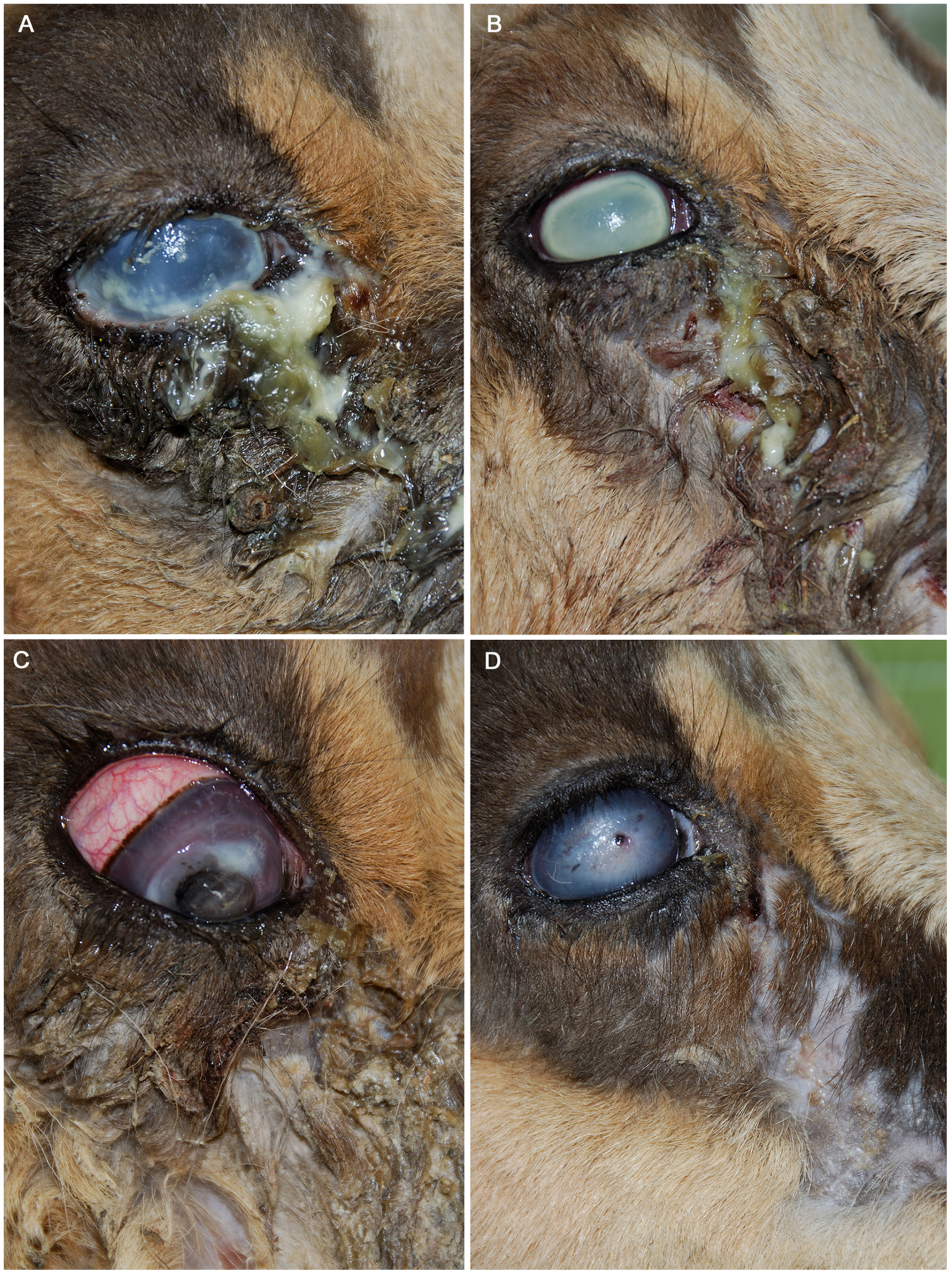
Stages lesion of IKC in Pyrenean chamois during 2006–2008 outbreak. Pictures show animals with different levels of disease severity**. A)** Purulent ocular lacrimation and mild corneal opacity (stage II). **B)** Corneal opacity (stage III). **C)** Late stage of IKC, with purulent exudation, evident conjunctivitis and corneal perforation (stage IV)**. D)** Animal without lacrimation that had chronic corneal lesions showing the face without hair.

All the affected chamois had lacrimation which ranged from serous to purulent. Three animals exhibited alopecia under the eye, but without lacrimation, and 91.4% (96/105) of the animals had bilateral macroscopic lesions. Individuals exhibited conjunctivitis and corneal opacity at various stages of lesion development. Based on a classification of ocular lesions [32], 26% (25/96) were at stage II or III and 63.5% (61/96) were at stage IV (e.g., severe lesions in the cornea such as perforation with iris protrusion) (Figure 7).

Two animals had unilateral lesions with corneal opacity and ocular effusion; the other eye was normal, but there were crusts on the hair below the eye. Three animals had been partially eaten by predators had both eyeballs missing, and four had one eyeball. Among the eyes that were present, three had ocular perforations and one exhibited corneal opacity. Three animals without lacrimation had chronic corneal lesions; one had bilateral corneal opacity in the vertex, and the other two had lesions that were characterized by an incarcerated iris in the cornea, and one exhibited corneal retraction.

**Figure 8:**
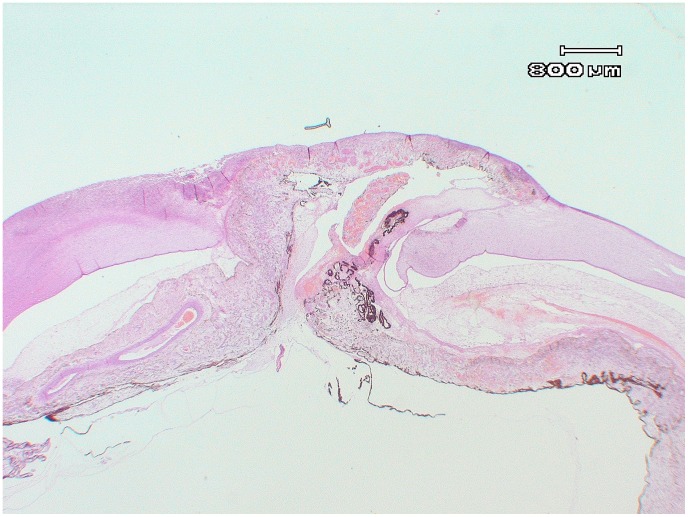
General view of the stage IV lesion of IKC in Pyrenean chamois. Note the ruptured Descemet’s membrane and synechia with the incarcerated iris and severe keratitis.

Animals in the early stages of the disease exhibited conjunctival hyperaemia, neutrophil and mononuclear cell infiltration, and lymphofollicular hyperplasia in the palpebral conjunctiva. Affected cornea exhibited inflammatory cells and vascular phenomena. In the advanced stages of the disease, the corneal epithelium was eroded, ulcerated, or perforated. Inflammatory cell infiltration and necrosis were present in the cornea. Most of the advanced cases exhibited ruptured Descemet’s membrane and synechia with the incarcerated iris (Figure 8). No lesions associated with IKC were found in the lung, liver, heart, kidney, or central nervous system tissues.

#### Mycoplasma and *Chlamydophila spp*. Detection

*M. conjunctivae* was detected in 93 (88.6%) out of 105 affected chamois. The age and sex of the infected chamois are shown in Table 3. In addition, *Mycoplasma spp.* was detected in three samples that were negative for *M. conjunctivae* (25%, 3/12). All the specific PCR assays used to detect *M. agalactiae* or the members of the *M. mycoides* cluster were negative. Fourteen samples tested positive for *Chlamydophila spp*. and 12 were positive for both *Chlamydophila spp*. and *M. conjunctivae*.

**Table 3:** Detection of *Mycoplasma conjunctivae* based on the ages and sexes of the animals checked (N = 105, 2006–2008).

Ages	Sex	Positive/tested (%)
Adults	Males	15/21 (71.5)
	Females	65/70 (92.9)
Yearlings/Kids	Males	4/5 (80)
	Females	9/9 (100)
**Total**		**93/105 (88.6)**

### IKC-non-affected Ruminants

Eight (4.2%) of the 190 chamois that did not have IKC signs, tested positive for *M. conjunctivae*. Among the animals sampled, prevalence was slightly, but not significantly higher during the outbreak (6.5%, 7/108) (95% CI: 2–11.15) than after (1.2%; 1/82) (95% CI: 0–3.6). None of the roe deer or red deer tested positive.

### Sheep

In 2008, among a sample of 349 animals from 17 flocks, *M. conjunctivae* was identified 102 times (29.2%; 95% CI: 24.5–34.3) and 15 flocks (82.3%; 95% CI: 56.6–96.2) tested positive. In 2009, among 268 animals from 15 flocks it was identified 57 times (21.3%; 95% CI: 16.5–26.7) and 13 flocks (84.6%; 95% CI: 54.6–98.1) tested positive. No significant differences were detected in the prevalence between years and between massifs. However, significant differences were observed between flocks (R^2^ Nagelkerke = 0.250; *p*<0.01).

### Management Actions

In the most affected GR (3) and massifs (8), hunting was banned until new population estimation was performed after the end of the outbreak. A small hunting quota (<1% extraction) was given to hunting ground owners (municipalities and communal owners) in two of the GR because of the economic importance of hunting to local communities, until a new population estimation was performed in the following year.

## Discussion

During the 2006–2008 IKC-outbreak, the spread of the disease was continuous and did not recur in the areas where it had already occurred, affecting a significant number of Pyrenean chamois and killing many animals. Recovery of the population was spontaneous. In most cases, reductions in infections and mortality paralleled the arrival of cold temperatures (a seasonal pattern), although a mild winter allowed the disease to persist until spring (no seasonality), as described in the Alps [5], [33].

In this outbreak, the distribution of dead animals among age classes did not differ significantly from the population at large, but adult females experienced significantly higher mortality than did adult males. Other studies have also found that mortality is biased toward adult females, but did not report the age-sex class structure of the population [5], [33].

The reduction of size of the population was similar to IKC responses in the Alps [5], [6], [13], [34]. It has been suggested that the mortality rates in subsequent outbreaks of IKC are lower than in the first outbreak [35]. We suspect that the 2006–2008 outbreak produced less mortality than the outbreaks in the late 1970s and the early 1980s in the Aragonese Pyrenees. The recovery after the outbreak showed an important annual increase, much larger than the pre-outbreak period.

In 2006–2008, *M. conjunctivae* was the main causal agent of the IKC outbreak, as reported in affected Pyrenean chamois in the Central Pyrenees, Spain [7]. The results also support the role of *M. conjunctivae* as the main aetiological agent of IKC in wild *Caprinae* [5], as previously reported in the Alps [4]–[6]. *M. conjunctivae* was common in all samples tested and despite the poor condition of some samples (e.g., partially eaten, frozen or in advanced stages of lesion), which underscored the benefits of the rt-PCR test for the routine diagnosis of *M. conjunctivae* [25]. However, the detection of this microorganism proved negative in 12 samples. It is possible that some or all of these were false-negatives because they were taken from animals in an advanced state of autolysis and, in three cases, with chronic corneal lesions. The lesion may suggest that the agent was no longer present, but other studies have demonstrated the temporary persistence of *M. conjunctivae* in the eyes of experimentally infected Alpine ibex [36]. Secondary bacterial infections of the conjunctiva and eye, contamination during sampling and extended period storage before analysis might have led to the absence of mycoplasma, which increases the difficulty of monitoring IKC in wild populations [7].

As in recent studies in Alpine chamois [37], in our study, mixed infections were associated with the outbreak of the Pyrenees. *Mycoplasma* and *Chlamydophila spp*. were found together in a significant number of animals, which indicated a possible occasional presence [38] or confirmed previous reports suggesting *Chlamydophila spp*. as a causal agent of IKC [10], [11]. *Chlamydophila spp*. was considered the most important species associated with IKC in the Pyrenees [16], although this might be because of the unusual difficulties involved in detecting a fastidious microorganism such as *M. conjunctivae*. Furthermore, in our study, *Chlamydophila spp*. was detected alone in two animals, which suggests either that it was an IKC infection caused by this bacterium only, or *M. conjunctivae* disappeared before the samples were analysed.

In addition, we detected non-identified mycoplasmas in conjunctiva samples, which had been found in Iberian wild goat *Capra pyrenaica* [39]. Other mycoplasma species such as *M. arginini*, which have been detected in the conjunctiva of other wild and domestic ruminants [39], [40], might have been present in the animals examined, usually associated with mixed infection.

Some mycoplasma species are known to cause keratoconjunctivitis in domestic small ruminant populations; such as *M. agalactiae*, and *M. mycoides* cluster members. Those species have been found in the conjunctiva of some wild ruminants such as the Iberian wild goat [39]. Other authors [41] have reported that many Iberian wild goats have been infected by *M. agalactiae* in southern Spain, which suggests that there is the potential for interspecific transmission between domestic and wild ruminants in that area. Those species were not agents of the IKC outbreak in Pyrenean chamois, which suggested a different epidemiological role of the various wild ruminants as carriers of contagious agalactia causing agents.

Some of the male samples were in poor condition or in advanced stages of lesion, which made it difficult to identify the agent. Field observations demonstrated that females and juveniles in herds were easier to detect by human observers than were male chamois, which are solitary for most of the year [42], [43]. In our study, most of the chamois examined were adults, and infections were biased toward females. In some cases, the largely solitary life of adult males might help them to eliminate the infection because the infection period in wild *Caprinae* appears to be temporary [6], [36], and does not exceed 6 months in sheep [44]. This behaviour could help avoid re-infections. As in populations of other wild Alpine *Caprinae*, the prevalence of *M. conjunctivae* in healthy Pyrenean chamois carried was low [45].

Early lesions were observed less frequently during the histopathological study. No animals were found at stage I because the game rangers documented only those animals that had evidence of blindness, which is apparent only when the ocular lesions are in an advanced stage [32]. No lesions associated with IKC were found in the histological sections of encephalon, which was previously described in Alpine chamois affected with IKC [46]. The histopathological lesions found in the eyes might not have been associated with a specific agent because *Chlamydophila spp*.- and *M. conjunctivae*-positive animals did not differ [1], [32].

Sheep farmers did not encounter more cases of IKC in the years of the outbreak (2006–2008); as also occurred in IKC-affected bighorn sheep foraged among domestic goats (Arizona, USA), which did not exhibit ocular signs [47], however, during outbreaks in mule deer in Utah, USA, and in chamois in the Central Pyrenees, Spain, cattle and, domestic sheep and goats, were affected, respectively [11], [15].

In the Suisse Alps, IKC persists in sheep, but not in chamois, being endemic in some areas. The transmission occurred in shared pastures between infected sheep and chamois, through flies and wind [2]. The specific dynamics in Pyrenean chamois outbreak might indicate that flies are more actives in warm season and probably imply more risk than wind to spread the disease.

IKC outbreaks in sheep [40] and wild ruminants have been linked to the introduction of new animals that did not have ocular signs, but were carriers of *M. conjunctivae* [47]. In our study, livestock were not affected by *M. conjunctivae,* which might suggest their involvement in the outbreak as asymptomatic carriers and that other factors were involved at the onset of the outbreak [48].

Our study demonstrates that *M. conjunctivae* is widespread among grazing sheep in all of the GR of the Aragonese Pyrenees; however, the persistence of infection in chamois is sporadic. Interactions with domestic sheep and other factors might have initiated the infection in chamois and its spread to other areas by the movements of infected animals. Not all of *M. conjunctivae* strains carried by sheep are transmitted to chamois or cause IKC [49]; therefore it is necessary to determine which of these strains were responsible for the outbreak.

In the Pyrenees, despite the restrictions on hunting rights and the negative economic impact, local hunters and municipalities agreed on a hunting ban. This was possible because of the strong implication of these interest groups in the management of the Pyrenean chamois population, specifically, through their participation on the advisory boards of the GR.

Long-term monitoring of the Pyrenean chamois population provided a basis for understanding the effect of the outbreaks, which improved management decisions, particularly important in a species subjected to extractive exploitation.
